# Heterotaxy Syndrome With Right Isomerism and Interrupted Inferior Vena Cava: A Case Report and Literature Review

**DOI:** 10.7759/cureus.55698

**Published:** 2024-03-07

**Authors:** Reema A Iskafi, Yazan Abugharbieh, Ibtihal Ahmad, Hidaya Shweki, Hisham A Dwaik

**Affiliations:** 1 Medicine, Palestine Polytechnic University, Hebron, PSE; 2 Radiology, Al-Ahli Hospital, Hebron, PSE; 3 Pediatric Cardiology, Al-Ahli Hospital, Hebron, PSE

**Keywords:** asplenia, interrupted ivc, left isomerism, right isomerism, heterotaxy syndrome

## Abstract

Heterotaxy syndrome (HS) occurs in developing embryos due to an inability to establish the normal anatomy, which manifests as abnormal symmetry and malposition of the thoracoabdominal viscera and vasculature, including cardiac and extracardiac anomalies. It is classified as right or left atrial isomerism. This classification depends on the atrial appendage morphology and the extracardiac defect associated with it. Right isomerism usually presents with right atrial appendages (RAA), asplenia, total anomalous pulmonary venous return, and severe pulmonary stenosis. In contrast, left isomerism usually presents with left atrial appendages, polysplenia, and an interrupted inferior vena cava (IVC). The interrupted IVC feature has never been reported with the right isomerism. Diagnosis of HS may take place prenatally or a few days postnatally due to the severe cardiac defect, whereas a left isomerism diagnosis may be delayed until adulthood. Despite the popularity of the HS classification, we reported a rare presentation of an interrupted IVC, dextrocardia, a right-sided aortic arch, and a total anomalous pulmonary venous return, which occurred along with the right isomerism major components (asplenia syndrome).

## Introduction

Congenital heart disease (CHD) and its complications are considered one of the leading causes of death in neonates, and the reported global prevalence continually increases to reach a maximum of 9.4/1000 between 2010 and 2017. One of the most complex CHDs is heterotaxy syndrome (HS) [1].

HS or isomerism is defined as an abnormality in the arrangement of internal thoracoabdominal organs across the left-right axis of the body. It is synonymous with visceral heterotaxy and situs ambiguous [2,3]. The term situs-ambiguous differs from situs-solitus, which is the typical positioning of the cardiac structures, and the rest of thoracoabdominal organs, or situs-inversus, which is the mirror image of situs-solitus. Situs-ambiguous is the intermediate presentation between both [3,4].

HS is characterized by multisystem affection, including cardiovascular, respiratory, and gastrointestinal systems. That explains why it is associated with high morbidity and mortality rates [5,6].

It's often categorized into two groups: right atrial isomerism (RAI) and left atrial isomerism (LAI). Isomerism describes the situation in which the morphologically right or left structures are duplicated and symmetrical (i.e., present on both sides of the body) [3,4].

In cases of left-atrial isomerism (polysplenia syndrome), the main feature is the presence of two morphological left atrial appendages. Also, this condition is characterized by bilobed lungs with hyparterial bronchi, polysplenia, and biliary atresia. In about 75% of cases, the inferior vena cava (IVC) is interrupted with azygous continuation. The hepatic veins, either isolated or in confluence, drain into either atria. Cardiac anomalies associated with LAI include atrioventricular septal defects, such as partial atrioventricular septal defects (partial AVSD), left-sided obstructive lesions (aortic stenosis, mitral stenosis, and coarctation of the aorta), and bilateral absence of the sinoatrial (SA) node. Double outlet right ventricle (DORV) and transposition of the great arteries (TGA) are rare. On the other hand, right-atrial isomerism (Ivemark syndrome) is characterized by two morphologically right atrial appendages, asplenia, bilateral trilobed lungs with periarterial bronchial trees, a central liver, bilateral superior vena cava, and intestinal malrotation. The IVC drains into one of the atria, and the hepatic veins may drain separately into the contralateral atrium. Cardiac anomalies associated with RAI include dextrocardia, univentricular atrioventricular connection, a common atrioventricular valve with complete atrioventricular septal defect, total anomalous pulmonary venous return, double inlet ventricle, and pulmonary stenosis or atresia. In contrast to LAI, TGA, and DROV are more commonly observed in RAI [4,5,7,8].

In this paper, we report a case of right-sided isomerism with interrupted IVC, infracardiac total anomalous pulmonary venous return, dextrocardia, and right-sided aortic arch in a one-week-old newborn, who presented with severe respiratory distress.

## Case presentation

A female term newborn presented with severe respiratory distress one week after her normal vaginal delivery. She had fetal echocardiography (ECG) at the 34th week of gestation which revealed an unbalanced atrioventricular septal defect along with a DORV and malposed great arteries which were highly suggestive of HS. Genetic studies (e.g., fluorescence in situ hybridization, cytogenetics, heterotaxy microarray) weren't done.

Her oxygen saturation (O_2_ sat) at birth was low (the exact value was not reported) so she was intubated for one day and then weaned to room air with O_2_ sat > 85%, blood pressure (BP) was stable, heart rate was within the normal limit and septic work-up was negative. The post-delivery ECG examination had similar findings to the fetal ECG report.

After one week, she had severe respiratory distress with O_2 _sat 65% which was elevated to 80-90% by bilevel positive airway pressure. The laboratory results were unremarkable.

**Figure 1:**
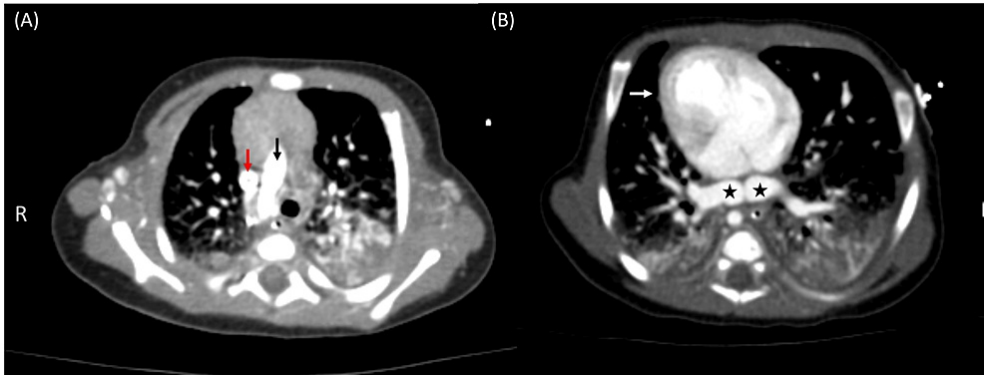
(A) Axial CT scan at the upper part of the chest shows a right-sided aortic arch (black arrow) and a normal right SVC (red arrow). (B) Axial CT scan at the level of the chest: a dextrocardia (white arrow) with abnormally dilated bilateral inferior pulmonary veins (stars). CT: Computed tomography; SVC: Superior vena cava

**Figure 2:**
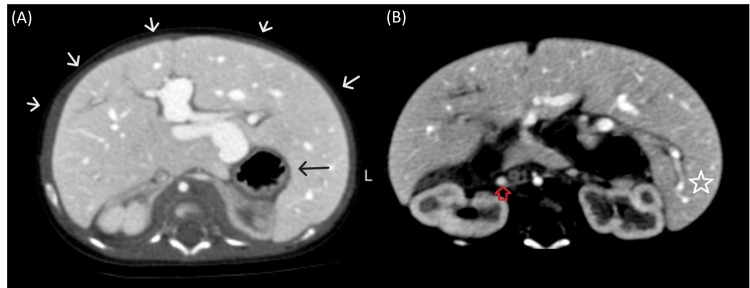
(A-B) Selected axial CT scan cuts at the level of the upper abdomen reveal a midline liver (white star and arrows), asplenia, left stomach (black arrow), and interrupted IVC (red arrow). CT: Computed tomography; IVC: Inferior vena cava

**Figure 3:**
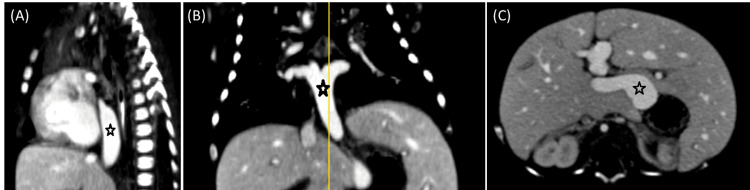
Selected coronal, sagittal, and axial CT scan cuts at the level of the chest (A-B) and upper abdomen (C) show an infracardiac total anomalous of pulmonary venous return communicating with the portal vein (star). CT: Computed tomography

Upon that, a chest X-ray and CT angiography (CTA) scan were ordered. The X-ray showed pulmonary congestion; the CTA revealed a right-sided aortic arch (Figure 1A), dextrocardia (Figure 1B), midline liver, left-sided stomach, and asplenia (Figure 2) compatible with right isomerism. Moreover, the pulmonary veins were communicating with an infra-cardiac isolated vein that drains directly to the dilated portal vein without evidence of left atrial drainage, suggesting an infracardiac total anomalous pulmonary venous return (Figures 1, 3). The IVC was interrupted (Figure 2B). The right and middle hepatic veins were draining directly to the right-sided atrium and the left hepatic vein was draining to the left-sided atrium. The superior vena cava was unilateral on the right side (Figure 1). The azygos and hemiazygos veins were very small in diameter. The ascending and descending aorta, the aortic arch branches, and the pulmonary artery (PA) were normal.

**Figure 4:**
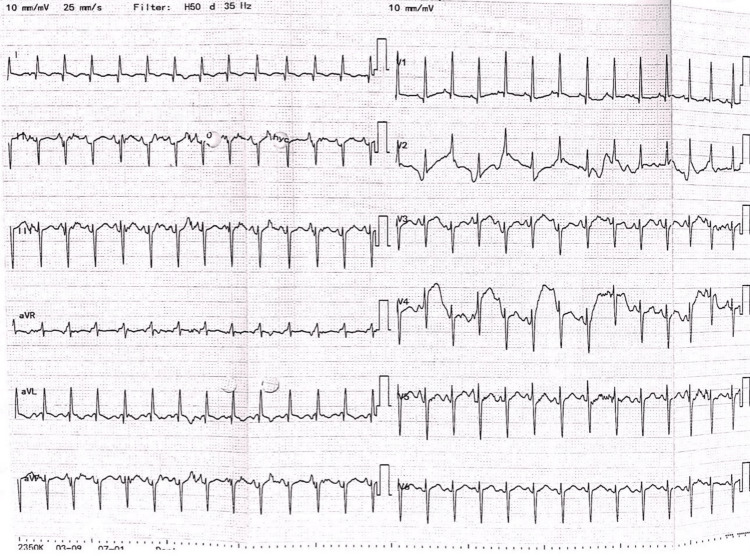
12-lead electrocardiogram shows sinus rhythm (leads II, V1, V6).

Abdominal ultrasound revealed a normal liver with a dilated intrahepatic biliary duct and mild right hydronephrosis. Transfontanel ultrasound was unremarkable. ECG showed sinus rhythm without significant abnormal findings (Figure 4).

The decision was made to proceed with open PA banding as a means of repair. Prior to the banding procedure, the patient's BP was 95/35 (68) mm Hg, the pulmonary artery pressure (PAP) was 97/45 (66) mm Hg, and the O_2_ sat level was 89% on 50% FiO_2_. Following the completion of the procedure, the patient's BP became 100/41 (63) mm Hg, the PAP decreased to 37/19 (27) mm Hg, and the O_2_ sat maintained at 75% on 40% FiO_2_.

In the pediatric intensive care unit, her oxygen saturation remained at approximately 75% while on a 1-liter nasal cannula for a week. Over the following three weeks, she experienced respiratory distress and bradycardia on four occasions, each time being managed based on the laboratory findings until the last instance when she also developed metabolic acidosis and hypotension. Despite receiving comprehensive medical support, she unfortunately passed away at 43 days old.

## Discussion

HS is a condition that arises during embryonic development when there is a disruption in the establishment of normal left-right asymmetry. This results in malposition of the cardiac, vascular, and abdominal viscera, as mentioned earlier.

The age of HS diagnosis varies depending on the extent and severity of cardiac involvement. Some patients get diagnosed prenatally or a few days after birth, while others may be diagnosed incidentally with unrelated symptoms in adulthood [5,6]. Most right isomerism cases are diagnosed prenatally by echocardiography because the cardiac involvement is more prominent and severe. However, prenatal diagnosis doesn’t improve the outcome [9,10]. Postnatally, various imaging techniques are used to diagnose both right and left isomerism, including cardiovascular magnetic resonance, CT, electrocardiography, and echocardiography, all of which to demonstrate cardiac vasculature and morphology and abdominal viscera alignment. Mild and wide ranges of cardiac involvement in left isomerism delayed the diagnosis. They may require no surgical intervention [11].

The most commonly used classification for HS into right and left isomerism depends on the atrial appendages morphology and extracardiac manifestation [9]. However, newly published studies thought that classification is "a mere statistical association and not a rule," as new cases reported to have features not related to only one isomerism but may include components of both [4,9,12].

Our case presented a disharmonious pattern between right isomerism (mostly) and left isomerism features; thus, we compared these features to the most commonly reported features of both right and left isomerism (Table 1) [5,9].

**Table 1:** Our case features compared to the most prevalent cardiac and gastrointestinal characteristics of left and right isomerism. (1) D-TGA: Dextro-transposition of the great arteries (2) PAPVR: Partial anomalous pulmonary venous return (3) TAPVR: Total anomalous pulmonary venous return (4) The patient passed meconium in the first 24 hours and didn’t complain of bilious emesis, abdominal distention, or any other finding suggesting malrotation. AVSD: Atrioventricular septal defects; SVC: Superior vena cava; GIT: Gastrointestinal tract; VT: Ventricular tachycardia

Structure	Most prevalent features of left isomerism	Most prevalent features of right isomerism	Features of our case
Cardiac			
Position	Levocardia	Mesocardia/dextrocardia	Dextrocardia
Atrial appendages	Left atrial appendages	Right atrial appendages	Right atrial appendages
Atrioventricular septum	Intermediate AVSD	Complete AVSD	Transitional AVSD
Ventricular morphology	Two good-sized ventricle	Single right ventricle with a hypoplastic left ventricle	double outlet right ventricle with hypoplastic left ventricles
Conduction system	Absent sinoatrial (SA) node > slow atrial or junctional rhythm and abnormal P wave axis, Complete heart block	Duplicated SA node > Atrial flutter, atrial tachycardia, junctional tachycardia, and VT	Single SA node > sinus rhythm
Vascular			
SVC	Bilateral (66%)	Bilateral (71%)	Unilateral on the right side
IVC	IVC interruption (75%)	IVC interruption has never been observed	IVC interruption
Great arteries	Normal	Malposed	Malposed (D-TGA) (1)
Pulmonary outflow	Unobstructed Pulmonary outflow	Severe pulmonary stenosis or atresia	Normal pulmonary outflow
Pulmonary venous return	PAPVR (2) Drain directly into the ipsilateral atrium	TAPVR (3)with an extracardiac communication. (supracardiac to the SVC or infracardiac to the portal vein)	TAPVR with an infra-cardiac isolated vein that drains directly to the dilated portal vein
GIT and hepatobiliary			
Spleen	Polysplenia	Asplenia or hyposplenia	Asplenia
Liver	Bilobed may be symmetrical	Central, transverse, and symmetrical	Central and transverse
Biliary tract	Biliary atresia	No abnormality	Dilated intrahepatic duct
Stomach	Right-sided	Near midline	Left-sided
Bowel malrotation	Less frequent	Less frequent	Not documented (4)

Right isomerism usually presents with total anomalous pulmonary venous return and severe pulmonary stenosis; interrupted IVC usually occurs in patients with polysplenia and left isomerism (75-80% of left isomerism); it was identified once with asplenia. However, it was never observed with right isomerism [5,13,14]. That makes our case the first of its kind, as an interrupted IVC, dextrocardia, right-sided aortic arch, and infracardiac total anomalous pulmonary venous return were presented with right isomerism (right atrial appendages and asplenia).

PA banding was done as the patient has dextro-transposition of the great arteries, a hypoplastic left ventricle, and an unbalanced AVSD in a way to reduce the pulmonary blood flow and to allow the ventricle to adapt to higher pressure for further repair [15].

Morbidity and mortality rates differ according to the patient's cardiac and extracardiac involvement. Cardiac congenital anomalies generally increase the morbidity associated with respiratory infections, which then leads to an increased mortality rate during the first year of life compared to patients who haven't developed pulmonary infection or with non-critical CHD [16]. Absent spleen increases the risk of infection with encapsulated organisms, sepsis, and the mortality rate compared to patients with polysplenia (85% to 50%, respectively) [9,17], so they require prophylactic antibiotics and vaccination.

HS can happen in isolation or may have predisposing factors, such as genetic factors (sporadic or inherited mutations mainly observed in left isomerism HS) and non-genetic factors (family history, maternal diabetes) [9]. Even though this baby had an antenatal diagnosis similar to the postnatal echocardiologic finding, it did not significantly impact her lifespan or prognosis. Therefore, genetic counseling could play a crucial role in preventing further cases of this syndrome.

## Conclusions

HS is a rare abnormality of visceral asymmetry with a wide spectrum of anomalies. It is characterized by multi-system involvement. The presented case has features of right isomerism and interrupted IVC. The latter is consistent with left isomerism. More research is required to consider redefining the used system of nomenclature and classification to include a combination of both right and left isomerism and broaden our awareness of this complex entity.
